# Adapting implementation science for higher education research: the systematic study of implementing evidence-based practices in college classrooms

**DOI:** 10.1186/s41235-020-00255-0

**Published:** 2020-11-05

**Authors:** Raechel N. Soicher, Kathryn A. Becker-Blease, Keiko C. P. Bostwick

**Affiliations:** 1 School of Psychological Science, Oregon State University, 2950 SW Jefferson Way, Corvallis, OR 97331 USA; 2 University of New South Wales, Sydney, Australia

**Keywords:** Implementation science, Translation, Higher education, Teaching, Learning, Use-inspired basic research

## Abstract

Finding better ways to implement effective teaching and learning strategies in higher education is urgently needed to help address student outcomes such as retention rates, graduation rates, and learning. Psychologists contribute to the science and art of teaching and learning in higher education under many flags, including cognitive psychology, science of learning, educational psychology, scholarship of teaching and learning in psychology, discipline-based educational research in psychology, design-based implementation research, and learning sciences. Productive, rigorous collaboration among researchers and instructors helps. However, translational research and practice-based research alone have not closed the translation gap between the research laboratory and the college classroom. Fortunately, scientists and university faculty can draw on the insights of decades of research on the analogous science-to-practice gap in medicine and public health. Health researchers now add to their toolbox of translational and practice-based research the systematic study of the process of *implementation* in real work settings directly. In this article, we define *implementation science* for cognitive psychologists as well as educational psychologists, learning scientists, and others with an interest in use-inspired basic cognitive research, propose a novel model incorporating implementation science for translating cognitive science to classroom practice in higher education, and provide concrete recommendations for how use-inspired basic cognitive science researchers can better understand those factors that affect the uptake of their work with implementation science.

## Significance statement

Originating to solve the research-to-practice gap in medicine, implementation science can make use-inspired basic research more useful in higher education. In this tutorial review, we define *implementation science* for cognitive psychologists with an interest in use-inspired basic research, propose a novel model incorporating implementation science for translating cognitive science to classroom practice in higher education, and provide concrete recommendations for how use-inspired basic cognitive science researchers can work with higher education instructors to boost college student academic achievement and learning.

## Introduction

Despite the tremendous advances in basic and applied research in cognitive psychology, educational psychology, and the learning sciences, few evidence-based practices have been taken up by college professors into routine practice in college classrooms, while ineffective practices stubbornly remain (Chew et al. 2018; Daniel and Chew 2013; Dunlosky et al. 2013; Dunlosky and Rawson 2019; Halpern and Hakel 2003; Kirschner and van Merriënboer 2013; McDaniel et al. 2007; Pashler et al. 2008; Roediger and Pyc 2012; Sumeracki et al. 2018). To help address this “research–practice gap,” the current tutorial has three aims: (1) introduce cognitive scientists (specifically those with an interest in higher education outcomes) to the interdisciplinary field of implementation science, which originated in public health and has grown quickly, (2) propose a framework to summarize the current research in cognitive science (broadly speaking) that supports evidence-based practice and integrates implementation science into existing models of basic, applied, and translational research, and (3) provide concrete suggestions for researchers on how to get started with implementation science in their own programs of research.

## What is implementation science?

Implementation science is a recent addition to the literature that may be unfamiliar to cognitive scientists yet is a promising component to help researchers meet their goals of implementing scientifically sound teaching in real-world settings. Implementation science is defined as “the scientific study of methods to promote the systematic uptake of research findings into routine clinical practice…” (The Improved Clinical Effectiveness through Behavioural Research Group (ICEBeRG) 2006). In other words, implementation science, in the higher education context, is the study of the methods used to promote integration of research findings into classroom practice and educational policy (Matlock and Glasgow 2017). Because a psychological intervention is “done with people, not on people,…we need to understand where and when people…will be able to use it in their lives” (Walton and Yeager 2020, p. 224). Implementation science examines the contextual factors influencing uptake and use of an intervention, such as feasibility, fidelity, and sustainability (Blase et al. 2012; Proctor et al. 2011). Additional examples of research questions from an implementation science lens are: how feasible is it for a student or instructor to use an intervention, how well do instructors deliver the essential components of the intervention, and how long is an instructor able to continue using an intervention (Fixsen et al. 2005). In this way, the process of how instructors use, adapt, and continue or discontinue the use of an intervention is the subject of scientific inquiry alongside the question of how well the intervention works (Blase et al. 2012). Thus, the goal of implementation science is to generate knowledge that will provide guidance in adapting an evidence-based practice to a particular context to maximize its previously demonstrated effectiveness (National Academies of Sciences, Engineering, and Medicine 2016).

Implementation science is distinguished from effectiveness or applied research as it is focused on the factors that contribute to the uptake of an established and effective intervention, rather than the effectiveness of the intervention in a real-world setting (i.e., effectiveness research). It is distinct from the work of individual practitioners who make use of the scientific literature in making decisions about what is best for clients, patients, or students. Applied research, effectiveness research, and scholarly practice are all necessary for generating, evaluating, and implementing effective interventions, yet the research–practice gap persists. By using implementation science to also understand the contextual factors that support and attenuate intervention effectiveness, researchers can deliver more precise interventions with more consistent results.

The need for science specifically addressing how effective interventions move into regular use in practice was first identified in the healthcare sector. A review of nine specific medical practices previously shown to be widely effective (e.g., cholesterol screening) found that it took an average of 15.5 years to reach a 50% rate of use post-publication (Balas and Boren 2000). In public health more generally, it takes an estimated 17 years for 14% of basic research to become best practice (Green 2001, 2008). To address the slow, often incomplete translation of research to practice (Green 2008), medical and public health researchers now study, in addition to pure basic, use-inspired, or applied research, how, where, and with whom clinicians use new practices. This growing field of implementation science helps researchers and clinicians to systematically study the factors that lead to successful implementation of efficacious and effective practices, with fidelity, and at scale. Like any research field, implementation science has its own theoretical models and frameworks to guide empirical research (for a review, see Tabak et al. 2012). Over decades, in medicine and public health, the field of implementation science has grown to include multiple journals, funding mechanisms, and conferences.

More recently, implementation science has been applied in health services psychology. In clinical psychology, or behavioral health services more generally, the Teaching-Family model has been lauded by the American Psychological Association as an evidence-based practice for treating at-risk juveniles (https://www.teaching-family.org, 2019). Even after determining the efficacy of the program and establishing effectiveness in a few group homes, the success rate of implementing the program at scale was only 15% (Fixsen et al. 2010). Twenty years later, that number rose to 80%. The researchers attributed this improvement to their systematic collection of “implementation-related data,” such as methods for training skilled implementers, organizational supports for the integration of the intervention, and leadership (Fixsen et al. 2010, p. 437). In a review of the 792 Teaching-Family programs across the USA, the authors concluded that “having well researched procedures working well in a prototype program is a good place to start but it is not sufficient to assure replicability and implementation” (Fixsen et al. 2007, p. 106). In other words, implementation research played a critical role in the success of the program by identifying the core components of the program (essential for maintaining fidelity of the program) and highlighting facilitators of successful implementation (to increase the feasibility for practitioners implementing the program).

In school contexts, the Working Group on Translating Science to Practice (Division 16, School Psychology) recognized that implementation science is essential for improving the use of evidence-based interventions (Forman et al. 2013). Specifically, implementation science is particularly useful for school psychology because it can be used to map out barriers and facilitators of implementation in schools, isolate critical components of interventions, and/or identify how to adapt interventions to local contexts and understand diverse populations and systems in which interventions are implemented. This working group argued that high-quality intervention research must include systematic measurement of implementation in practice settings, something that can only be accomplished with implementation science (Forman et al. 2013).

## Applying implementation science to higher education

Some initial work has begun to apply implementation science to (primarily K-12) education. The *Handbook of Implementation Science for Psychology in Education* further illustrates that psychologists find implementation science to be central to bringing evidence-based practices to bear in educational settings (Kelly and Perkins 2012). Blase et al. (2012) noted that educational psychologists trained in implementation science have much to offer: expertise in the feasibility of using an intervention at the student, instructor, or institutional level, knowledge of the core components of evidence-based practices available needed to ensure fidelity, and the skills for assessing sustainability of the intervention over time.

Although initial work in implementation science in K-12 settings has offered important information on how to translate research to schools, more work is needed in the higher education sector specifically. Implementing cognitive science in higher education teaching and learning practices will differ from practices in K-12 in important ways. K-12 teachers are explicitly trained to teach in colleges of education, and are required to complete ongoing professional development, often determined by school administrators rather than themselves, to remain licensed. In contrast, instructors in higher education typically receive little to no formal training in teaching or learning and are not required to obtain any professional development training once on the job. In addition, the structure of class periods and terms, decision-making authority, accommodations for students with disabilities, and many other factors differ greatly between the two settings. Given the greater variability in instructor ability, instructor professional development, and course structures in higher education, there is a need for models of implementation science specific to higher education. Therefore, in this paper, we focus on translating cognitive science for teaching and learning specifically in higher education.

There are existing examples of implementation science at the crossroads of psychology, education, and the learning sciences, such as “Design-Based Implementation Research” (Penuel et al. 2011). The goal of design-based implementation research is the improvement of educational systems through coordination of interdisciplinary teams (researchers and community-based practitioners) to work on problems of practice, iterative design, development of implementation theory, and capacity to support sustainable changes. Unfortunately, the proponents of this field of research themselves acknowledge that implementation research in education has yet to find a home. They describe most of this research as “fugitive documents”—studies that are published through less formal networks (e.g., book chapters, conference presentations)—and stress the need for peer-reviewed journals in this area (Penuel et al. 2011). Identifying an implementation science framework within higher education provides design-based implementation research an intellectual home, supports further work in this area, and helps to facilitate the translation of research into practice.

In this tutorial review, we draw on the excellent headway that has already been made in closely related areas to help publicize and bolster additional support for the use of implementation science in higher education research. Current evidence suggests that (1) progress from laboratory to classroom (either K-12 or higher education) is slow and uneven (e.g., Weinstein et al. 2018), (2) implementation science is a viable option for speeding the adoption and scaling up of effective interventions (e.g., Fixsen et al. 2005), and (3) a specific and sufficient infrastructure does not yet exist to support implementation science (e.g., Penuel et al. 2011). In the current tutorial, we build a framework for moving research into practice in higher education, offer suggestions for how researchers can get started with implementation science, and identify potential barriers to using implementation science in higher education.

## A framework for moving research into practice in higher education

**Fig. 1:**
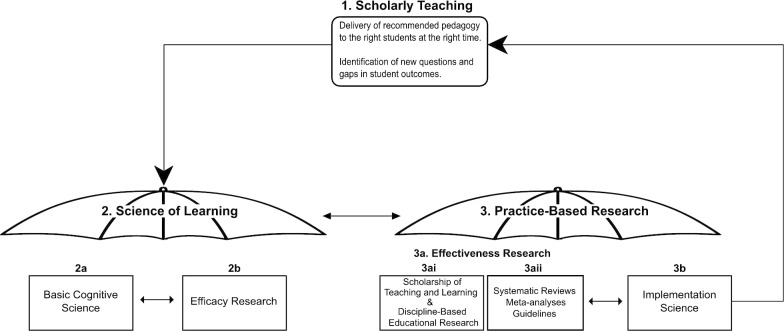
A framework for moving research into practice in higher education

Because many strong efforts are underway to bring scientifically sound teaching to real-world settings, it is helpful to organize these efforts and differentiate them from implementation science. Figure 1 represents a potential framework for synthesizing the myriad types of cognitive science that contribute to evidence-based practices and/or pedagogy in college classrooms, of which implementation science, we argue, should be a part. Notably, this framework assumes that the goal of science in this context is evidence-based practice (also called “scholarly teaching” and analogous student practices that might be called “scholarly learning,” consistent with the health literature where the outcome variable is the provision of evidence-based health care). The framework consists of three major sections, including scholarly teaching, science of learning, and practice-based research, and acknowledges that this research system is nonlinear and iterative. The framework highlights how key areas work together to help move research into practice in higher education.

**1. Scholarly teaching.** The goal of translating cognitive science is to support instructors in scholarly teaching. Scholarly teaching involves (1) identifying a problem in the classroom, (2) documenting baseline behavior, (3) investigating what others have done to address the same problem, (4) selecting, with justification, an intervention to improve student outcomes, (5) systematically observing and recording the application of the intervention, and (6) comparing results to baseline (Richlin 2001). Selection of an intervention is an especially important step; we argue that implementation research supplements findings from use-inspired cognitive research, as it identifies and examines essential contextual information instructors need to decide whether a pedagogical practice is likely to be effective *in their specific setting*. Scholarly teaching is achieved when instructors apply scientific knowledge about teaching and learning to their pedagogical practice and the assumption is that this form of teaching would lead to improved student outcomes (e.g., learning). Additionally, instructors help to identify new problems that can feed back to laboratory-based research.

**2. Science of learning.** Within the realm of cognitive science, the Science of Learning encompasses both basic cognitive science and efficacy research, with an emphasis on the application of cognitive theory to educational settings (Daniel and Chew 2013; Rowland 2014; Science of Learning, National Science Foundation n.d.).

**2a. Basic cognitive science**. Basic research under highly controlled conditions is essential for defining causal mechanisms and the size and strength of a relation between variables. Cognitive scientists may use this type of research to gain an understanding of how cognitive processes like learning, memory, and attention work. Results of this type of research form the theoretical foundation upon which potential interventions to alter or improve these mechanisms are based. Additionally, the research efforts in the basic cognitive science realm can be informed by instructors’ experiences to help direct research agendas most likely to be later integrated into classroom practice.

**2b. Efficacy**. Efficacy research is used to evaluate the impact of an intervention under ideal—usually laboratory—conditions (Flay 1986; Flay et al. 2005; Rabin and Brownson 2017; Sawyer and Dunlosky 2019). The intervention is typically one that was identified through basic cognitive science, with a known or hypothesized theoretical causal mechanism. The hallmark characteristics of efficacy research are random assignment of participants to groups, single- or double-blind design, and a control group that represents the current standard practice. Cognitive scientists conducting use-inspired basic research are an example of efficacy research. For example, use-inspired efficacy research in higher education classrooms may use authentic learning materials and/or assessments. This type of research is critical for illustrating the types of pedagogical practices and/or study strategies that researchers could evaluate in real-world settings (Flay 1986; Sawyer and Dunlosky 2019). Without this research, negative or null results associated with the practice in real-world contexts cannot be clearly interpreted. Null results could be due to something about the intervention itself or due to ineffective use of the intervention, and data are not typically collected that would help distinguish between these two options (Flay 1986; Sawyer and Dunlosky 2019).

**3. Practice-based research**. Practice-based research refers to research conducted in applied or real-world settings, including effectiveness research, reviews and meta-analyses, and implementation science.

**3a. Effectiveness research**. Practice-based effectiveness research investigates whether a practice/strategy/intervention, found to be efficacious in laboratory settings, does more good than harm in a real-world context (Flay 1986; Flay et al. 2005). Effectiveness research incorporates many of the rigorous methods of laboratory-based research, including random assignment of participants, single- or double-blind designs, and authentic materials and assessment. A large body of practice-based effectiveness research already exists in cognitive science. Funding mechanisms exist explicitly for this type of work in both K-12 and higher education settings (e.g., from the Institute of Education Sciences and the National Science Foundation). Practice-based effectiveness research is published in outlets such as the *Journal of Educational Psychology*, the *Journal of Research on Educational Effectiveness*, and *Learning and Instruction*. The results from effectiveness research elucidate whether a practice can improve learning, memory, or other education-related constructs with real students in actual classrooms. Positive results serve as evidence that the practice can be made available to students and delivered in a manner that is acceptable to them (Flay 1986; Rabin and Brownson 2017).

**3ai. The scholarship of teaching and learning**. One current approach to effectiveness research is the scholarship of teaching and learning. The scholarship of teaching and learning (SoTL) is defined as “the systematic study of teaching and learning, using established or validated criteria of scholarship, to understand how teaching (beliefs, behaviors, attitudes, and values) can maximize learning, and/or develop a more accurate understanding of learning, resulting in products that are publicly shared for critique and use by an appropriate community” (Potter and Kustra 2011, p. 2). In the SoTL, where the researchers are typically the classroom instructors, the researchers are interested in questions related to improving student outcomes in their own classrooms and also beyond them (Hutchings and Shulman 1999). Instructors who are also psychological scientists are especially well positioned to test interventions from the use-inspired basic science literature in their classrooms to see “what works.” The results of this research are typically published in peer-reviewed journals such as *Teaching of Psychology* or *Scholarship of Teaching and Learning in Psychology*.

Instructors engaged with the SoTL in disciplines other than psychology may also refer to their research as discipline-based educational research (Gurung et al. 2019). The goal of continuous improvement of classroom practice is a hallmark of both the SoTL and discipline-based educational research (Gurung et al. 2019).

**3aii. Reviews, meta-analyses, and guidelines.** As the scientific literature accumulates over time, researchers conduct systematic reviews and meta-analyses to provide guidelines and/or an overview of “best practices” for instruction in higher education. Sometimes, these syntheses even occur in the format of popular press books, where cognitive scientists write about effective practices for more general audiences (e.g., Agarwal and Bain 2019; Brown et al. 2014; Willingham 2009). These different forms of research syntheses can provide additional evidence of the effectiveness of different pedagogical practices or highlight practices/strategies/interventions that are likely to generalize across a range of real-world settings. These resources are part of strategic dissemination efforts to help inform classroom approaches (Dunlosky and Rawson 2019; Roediger and Pyc 2012). The results of these syntheses are important for informing instructors and for providing critical feedback to use-inspired basic researchers, who can use the information to inform future research directions.

Although research in the scholarship of teaching and learning (3ai) and reviews, meta-analyses, and guidelines (3aii) are well-supported, they are not without their challenges. For example, Mark Schneider, Director of the Institute of Education Sciences, noted that due to the limited scope of most funded studies that found an impact, it was impossible to judge if the interventions would work with different types of students or at different institutions (2018). Since then, funding mechanisms have begun to focus on replication projects with the aim of better identifying contextual factors related to successful interventions. Thus, although effectiveness research is relatively well established and offers important insights into how interventions function in applied settings, implementation science is still needed to understand how effects may vary across contexts.

**3b. Implementation science.** Implementation science is related to, but distinct from, higher education-relevant cognitive psychology (e.g., Dunlosky et al. 2013), science of learning (e.g., Halpern and Hakel 2003), educational psychology (e.g., Bernacki et al. 2019), SoTL (e.g., Corral et al. 2019), discipline-based educational research in psychology (e.g., National Research Council 2002), and the learning sciences (e.g., Sawyer and Dunlosky 2019). Implementation science builds on effectiveness research in applied settings—an existing evidence base is essential for designing implementation research studies (Lane-Fall et al. 2019). Implementation science is different from current research approaches in that, rather than trying to determine whether a pedagogical practice improves learning, it focuses directly on variables that lead to broad uptake of effective practices over time, such as fidelity, feasibility, and sustainability (e.g., Ford et al. 2017).

For example, Hulleman and Cordray (2009) examined the extent to which treatment fidelity affected participant motivation for a motivational intervention in both laboratory and classroom settings. They found that the achieved relative strength of the intervention in the classroom was significantly less than in the laboratory. Additionally, their research showed that the reduction in treatment fidelity was attributable to teacher factors (i.e., providing opportunities to complete the intervention) rather than student ones (i.e., completing the intervention as intended). As there is rather limited implementation science research in higher education settings, further below, we provide concrete recommendations for how cognitive scientists can get started with and contribute to this component of the scholarly teaching cycle.

## Summary

Overall, the description of the framework thus far organizes current research approaches meant to bring cognitive science to bear on classroom practice. All elements of the model are crucial—translation would not be possible without even one of them. Improved communication and/or collaboration between researchers in each area of the model is necessary, but not enough, to help instructors achieve scholarly teaching. To improve the adoption and scaling up of effective practices, the available evidence should include systematic assessment of the contexts and processes related to implementation of those practices. Table 1 is a summary of the terminology used in the framework accompanied by (necessarily limited) examples of each term.

**Table 1:** Summary of framework terminology with examples

Term	Definition	Example
Scholarly teaching	The application of scientific knowledge about teaching and learning to pedagogical practice (Richlin 2001)	Increased use of low-stakes or no-stakes practice testing in the classroom (as recommended by Dunlosky et al. 2013)
Science of learning	Research producing basic theoretical insights and fundamental knowledge about learning principles, processes, and constraints (NSF n.d.)	Changes to neural oscillations can improve multi-tasking performance (Hsu et al. 2017)
Basic cognitive science	Research that generates causal models to explain a phenomenon of interest (Sussman et al. 2006)	Repetition improves performance in visual and cross-modal, but not auditory, recognition tasks (Amir Kassim et al. 2018)
Efficacy research	Research to determine whether an intervention improves outcomes in a tightly controlled setting (Flay 1986)	Benefits from retrieval practice are greater for students with lower working memory capacity (Agarwal et al. 2017)
Practice-based effectiveness research	Research to determine whether an intervention improves outcomes in a real-world context	Classroom-based programs of retrieval practice reduce middle school and high school students’ test anxiety (Agarwal et al. 2014)
Scholarship of teaching and learning (SoTL)	Research designed to understand how teaching (beliefs, behaviors, attitudes, and values) can maximize learning (Potter and Kustra 2011)	Low performers respond positively to specific and general teacher feedback, while high performers have lower performance following specific, but not general, feedback (Baranczyk and Best 2020)
Discipline-based educational research	Discipline-specific scholarship of teaching and learning	Final exam scores for students in introductory physics courses can be predicted by math SAT (or ACT) scores and concept inventories (Salehi et al. 2019)
Systematic reviews, meta-analyses, and guidelines	Comprehensive summaries that synthesize a body of literature to make recommendations for practice	For researchers: Studies controlling for text difficulty and instructor variance are necessary for examining the effects of open versus traditional textbook us on students’ course performance (Griggs and Jackson 2017) For instructors: Specific advice for using evidence-based practices, such as retrieval practice and spacing, to improve teaching (Agarwal and Bain 2019)
Implementation science	Research that seeks to understand the processes and factors that are associated with successful integration of evidence-based interventions within a particular setting (Rabin and Brownson 2017)	Students who identify as both first-generation and underrepresented minority students benefit from a writing utility value intervention (Harackiewicz et al. 2016) The achieved relative strength of the utility value intervention is less in classroom studies than in laboratory ones, but still significant (Hulleman and Cordray 2009)

## Getting started with implementation science in higher education classrooms

Here, we make four concrete suggestions about how to begin using implementation science in teaching and learning research in higher education: (1) conceptualize instructors as scientist–educators, (2) use pragmatic-controlled trials in research designs, (3) adopt and adapt planning and evaluation frameworks to this field, and (4) expand and improve transparency of reporting. Together, we believe these to be important next steps in facilitating implementation science to translate cognitive science to practice. A summary of our recommendations for getting started with implementation science is presented in Table 2 and details of each recommendation can be found in the sections that follow.

**Table 2:** Summary of recommendations for getting started with implementation science

To get started	Explanation	Role of use-inspired basic cognitive science
Instructors as scientist–educators	Promote a scientist–educator model for instructors to encourage scholarly teaching	Working knowledge of cognitive science and scientific literacy is essential for educators; role for cognitive scientists as consultants
Design pragmatic-controlled trials	Use random assignment and blinded procedures to test the effectiveness of interventions in real college classrooms with an emphasis on generalizability	Use-inspired cognitive scientists needed to identify essential components; role for cognitive scientists as consultants
Use a planning and evaluation framework	Before conducting research, use a planning and evaluation framework to highlight naturally occurring moderator variables to examine within the research design If research has already been conducted, use a planning and evaluation framework to assess the extent to which the study design matched real-world settings	Cognitive scientists can collaborate with educators to identify potential moderators and design ways to measure them
Expand reporting of research	Systematically document issues related to exclusion/inclusion of settings (e.g., classrooms or universities), instructors, and students, reasons for exclusion/inclusion, and extended monitoring of the intervention after the project ends	Provides basic researchers, practice-based researchers, and scholarly instructors with shared terminology and standards, making research interpretable across settings and goals (e.g., were color-blind participants included?)

## Conceptualize college instructors as scientist–educators

At the center of implementation science is the concept of an evidence-based practitioner, the person who uses (or implements) scientific findings in a real-world context. This practitioner is acknowledged as having both the capacity to interpret the scientific literature and the practical experience needed to modify evidence-based recommendations for care (Palinkas 2019). In the same way, clinicians are thought of as evidence-based practitioners, instructors combine both basic cognitive science and knowledge of their discipline to examine their teaching, seek continuing education or professional development opportunities, and acknowledge that psychological science plays an important role in addressing educational problems (Newcombe 2002). To further support instructors as pedagogical experts, in addition to content experts, additional professional development is needed to ensure instructors (1) stay updated with advances and methodologies in teaching and (2) use methods of psychological science to empirically test the effectiveness of their teaching practices (Buskist 2013; Chew et al. 2018).

A useful model for understanding the role of the instructor in this translational framework is that of the “scientist–educator” (Bernstein et al. 2010). The idea of a scientist–educator comes from that of the scientist–practitioner from counseling, clinical, and school psychology (Petersen 2007; Shapiro 2002). A scientist–educator appreciates the complexities of excellent teaching and systematically collects evidence about their teaching effectiveness, reflecting on and using that evidence to revise teaching, and sharing what they have learned with peers. As a scientist–educator, one need not be an expert in research per se, but one must do “more than merely use others’ teaching techniques without evaluation of their effectiveness” (Bernstein et al. 2010, p. 30). The scientist–educator is interested not only in student learning, but in the interaction of many variables related to the instructor, the students, and the context in which they teach. More experienced instructors are most likely to identify which contextual variables are likely to influence interventions and it is these variables that implementation science aims to measure.

Conceptualizing instructors as scientist–educators puts instructors and researchers on the same level. When scientist–educators and researchers both contribute to the design, delivery, and examination of educational interventions, such interventions can harness both relevant science of learning research (that researchers may be more familiar with) and the complex contextual expertise of the instructor. Moreover, as the culture shifts to treat instructors more like scientists, collaborations between cognitive researchers and educators can grow and improve, like how community-based participatory research advances partnerships between researchers and knowledge users in other fields (e.g., Jull et al. 2017). Additionally, there is a role for cognitive scientists to host professional development workshops to help disseminate scientific knowledge relevant to educators and help de-implement ineffective practices, like matching instruction strategies to student learning styles (Pashler et al. 2008). Overall, conceptualizing instructors as scientist–educators has the potential to benefit multiple areas of teaching, learning, and research—including implementation science.

## Pragmatic-controlled trials

A major focus of implementation science is generalizability or external validity. Implementation science aims to understand the complex contexts in which the use of an intervention can be maximized. Thus, the research designs integral to implementation science systematically measure, rather than control, variables thought to be important moderators or mediators of interventions.

Although randomized-controlled trials (RCTs) are considered a gold standard in basic, use-inspired basic, and SoTL research (Wilson-Doenges et al. 2016), they tend to treat variables likely to influence the use and uptake of an intervention in the classroom, such as student’ backgrounds, class size, or selectivity of the institution as variables to be controlled. In other words, RCTs are considered “explanatory”—they show that an intervention can work under controlled conditions in a specialized population. In contrast, implementation studies are designed to work in “messy,” real-world settings. RCTs work to either control for or minimize the impact of confounding variables on outcomes through selection and randomization processes, whereas in implementation science, the particulars of those potentially relevant factors are central to the research questions being asked. If an intervention from an RCT is applied to a classroom and found to be effective, there were likely key adaptations made that would be important for other instructors to know about. If the application results in null effects, it will be difficult to know why. Because of this, there is not much known about how an intervention found to be efficacious or effective in an RCT would (or would not) be effective in other settings with other students.

One way to gain information about contexts (and therefore, facilitate implementation science) is to also conduct “pragmatic-controlled trials” (PCTs; Maclure 2009). PCTs are “pragmatic”—they are a real-world test in a real-world population. PCTs retain important hallmarks of RCTs in real classroom research—random assignment and blinded procedures—but emphasize issues of generalizability, including reach (who participated), adoption in diverse contexts, implementation methods, and sustainability of the intervention (Maclure 2009). By systematically measuring these differences across intervention sites, researchers are better able to identify contexts and populations where interventions work best, helping to answer questions of generalizability and external validity. While the goal of an RCT is to determine cause and effect, the goal of a PCT is to improve practice and inform decision-making, allowing for flexible protocols and local adaptations, and capitalizing on outcome measures that are easily collected and useful in everyday practice (for a review of PCTs, see Schwartz and Lellouch 1967; Thorpe et al. 2009).

In a study of higher education, researchers might use a PCT design to randomly assign students to a particular intervention, like in an RCT. In this case, the main outcome variable is the effectiveness of the intervention (e.g., exam scores). Additionally, the PCT design would use standardized, validated, and reliable quantitative measures to assess process outcomes like feasibility or acceptability of the intervention to instructors or students (Bywater 2012; Weiner et al. 2017). Results from those assessments might show, for example, that the intervention improved exam scores for students but that the instructors considered the intervention too burdensome to implement regularly in a course. PCTs often rely on qualitative methods to simultaneously monitor implementation of the intervention and gain insight into environments where the intervention was more successful (e.g., if the students saw greater gains under one instructor than another). Using PCTs to evaluate complex interventions is one aspect of a continually cyclical process of planning, evaluation, and revision to reach the most successful implementation possible.

## Planning and evaluation frameworks

Implementation scientists use planning and evaluation frameworks to provide a “systematic way to develop, manage, and evaluate interventions” (Tabak et al. 2012, p. 337). There are over 85 implementation frameworks listed at https://dissemination-implementation.org at the time of this writing. Frameworks help isolate essential components of interventions, leading to a greater understanding of when, where, and how interventions may work best.

To illustrate how to use a framework in higher education research, we start with the RE-AIM framework (Glasgow et al. 2019). Originally designed from a social-ecological perspective to enhance systems- and community-based research in public health policy, the RE-AIM framework identifies interventions that work in real-world environments by emphasizing the collection of evidence in dimensions beyond simply efficacy of the intervention (Glasgow et al. 1999). All five components of the RE-AIM framework are crucial for “evaluating programs intended for wide-scale dissemination” (Glasgow et al. 1999, p. 1325).

The main components of the RE-AIM framework are: Reach—individual-level participation and documentation of characteristics for both participants and non-participants, Efficacy—individual-level documentation of positive and negative outcomes, and behavioral outcomes for both students and instructors, Adoption—individual- and system-level characteristics of adopters and non-adopters, including barriers for non-participating settings, Implementation—system-level measurement of the extent to which an intervention is delivered as intended, and Maintenance—individual- and system-level measurement of long-term outcomes.

In a study of higher education, to assess the reach of an intervention, researchers would track the number of people excluded from the study, the number of people eligible to participate in the study, and compare the differences between those participating and those not on important variables. For example, the researcher might compare participating students’ socio-demographics to those of all students eligible to participate. This comparison not only provides greater detail about the individuals who did (and did not) participate but also gives more insight into how representative the participants are of the target population. RE-AIM also emphasizes, under efficacy, the tracking of negative (or unintended) outcomes, such as a finding that students who perform poorly on the first exam in the class ending up with lower final exam scores following the intervention than students who perform well on the first exam. As these examples show, the methodology does not explicitly differ from what researchers may already be doing, but provides tools to help researchers focus on external validity in both the planning and evaluation stages of a study.

## Expand reporting guidelines

To increase external validity, a central tenet of implementation science, reporting of all research (from basic to use-inspired to practice-based) should be expanded to systematically document issues related to exclusion/inclusion of settings (e.g., classrooms or universities) and instructors, reasons for exclusion/inclusion, and extended monitoring of the intervention after the project ends (Glasgow et al. 2019). The goal of expanded reporting is to understand the complex contexts in which the research occurs, with an emphasis on generalizability, transparency, and replicability (Glasgow et al. 2018). Clearer descriptions of informed consent procedures, which may affect rates and quality of participation (Kotz et al. 2019), and the specific core components thought to be essential for future implementation should also be reported (Michie et al. 2009; Moir 2018). Creating more detailed reporting guidelines helps to facilitate implementation science as it helps future researchers to identify the extent to which their context differs from previously studied samples and populations.

**Fig. 2:**
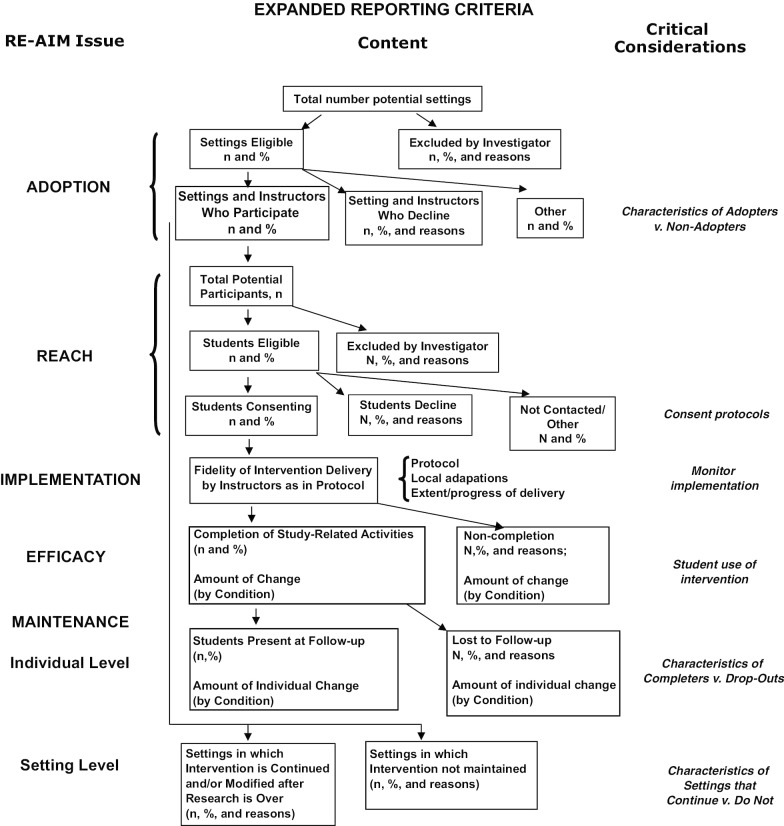
Expanded CONSORT diagram adapted to research in teaching and learning in higher education ( adapted from Glasgow et al. 2018)

One suggestion from implementation science for expanded reporting comes from the “Expanded CONSORT” guidelines (Glasgow et al. 2018). CONSORT guidelines were originally adopted as minimal reporting criteria for RCTs in medical research (Schulz et al. 2010). The suggested expansion, born from the RE-AIM framework, adds detailed information about participation and representativeness at all levels, including maintenance and sustainability after the study ends. This additional information could easily be incorporated as online supplementary materials submitted to journals with manuscripts. A diagram of expanded CONSORT reporting applied to teaching and learning in higher education is shown in Fig. 2.

## Expanded reporting and open science

While practices such as sharing open data and increasing transparency are relevant to all sub-fields of psychology, some have argued it is of particular importance for cognitive science and data-intensive fields (Paxton and Tullett 2019). Expanded reporting aligns well with open science practices (National Science Foundation and Institute of Education Sciences 2018). For example, increased transparency in reporting a larger range of methodological details is consistent with the recommendation that scientific journals in psychology require authors to “disclose all of their measures, manipulations, and exclusions, as well as how they determined their sample sizes” (Nelson et al. 2018, p. 518). Additionally, increased transparency aids in replication. Replication of a finding must result under “specifiable conditions” (Nelson et al. 2018, p. 520). Therefore, by definition, replication requires more detailed reporting of the context in which the research takes place.

Increased transparency—including preregistration of implementation studies—will promote the distinction in published studies between exploratory and confirmatory analyses (Nelson et al. 2018). Preregistrations are “time-stamped plans for data analysis written before any data are analyzed” (Nelson et al. 2018, p. 519; Wagenmakers et al. 2012). This level of transparency helps guard against questionable research practices such as p-hacking (Simmons et al. 2011) and HARK-ing (Kerr 1998). In implementation science, preregistration of studies should include detailed information about (1) predictions for and measurement of variables related to students (individual level), instructors (staff level), settings (classroom level), and systems (university/college level), (2) the implementation protocol to aid researchers in assessing fidelity of implementation, and (3) methods for gathering maintenance data beyond the end of the study protocol. A sample preregistration form for teaching and learning research using expanded reporting and a RE-AIM framework is available on the Open Science Framework project page (https://osf.io/kj83v).

## Potential barriers to using implementation science

Despite the advantages of implementation science and the potential of these methods to fill in parts of the research–practice gap, the use of implementation science is not a magic bullet. As researchers embark on the implementation science journey, it should be noted that there will be challenges to face. Indeed, this is one area in which efforts in other fields are most informative.

**1. Inconsistent terminology.** This challenge is twofold. The first is the lack of common terminology in research in higher education. The term “translation” has different meanings for different people. Daniel (2012) explains translational research as a series of steps from exploration in a laboratory setting to dissemination and continued refinement of a promising practice. Others, however, understand translation to refer specifically to design and dissemination of interventions appropriate for use by instructors. The second challenge is the lack of common terminology in the field of implementation science. National agencies in the USA refer to this field of research as implementation science, while Canadian agencies use the terms “knowledge transfer” or “knowledge translation” (Rabin and Brownson 2017). Success in adopting implementation science to address questions in higher education will require some flexibility on the part of researchers as well as concerted efforts to define the research process in this context.

**2. Funding.** In health fields, national funding agencies have embraced implementation science and developed grant funding specifically for those projects (e.g., National Heart Lung and Blood Institute and National Institutes of Health 2011). Even so, it is estimated that only about 10% of total grant expenditures in those areas are earmarked for implementation research (Colditz and Emmons 2017). Meanwhile, in education research, funding is more difficult to come by. Notably, the Institute of Education Sciences offers Development and Innovation grants which could fund some implementation work (Buckley and Doolittle 2013). However, these grants are also designed to fund development of an intervention or test innovations of an existing program, which are not implementation research per se. Previously, the National Science Foundation funded implementation research through the Research and Evaluation on Education in Science and Engineering Program competition, but this has been replaced by a new program with an emphasis on fundamental research. Per the program description, the “implications of funded projects for practice is likely to be long-term and indirect, influencing other, intermediate research literatures before affecting practice” (National Science Foundation 2019).

**3. Challenges with cross-institutional and international research.** Successful application of implementation science in higher education will require research to occur in a wider range of settings than predominantly white, research-intensive universities. Indeed, the focus on generalizability is a strength of implementation science as a field. However, access even to community college and minority-serving institutions (e.g., Hispanic-serving institutions and/or Historically Black Colleges and Universities) is hampered by (both perceived and actual) logistical challenges with institutional review boards (IRBs), faculty turnover, and incentives that do not universally support this kind of research. These issues are compounded with international collaborations, essential to extend this research outside of Western cultures (e.g., Henrich et al. 2010). Institutions, IRBs, and funding agencies can help researchers break down these barriers.

**4. Researcher–educator partnerships.** Researchers are not only challenged by barriers within (and across) institutions, but also by creating, building, and maintaining community partnerships. Within implementation science, researchers rely on community-based participatory research approaches to recognize the importance of stakeholder participation in the research process (Minkler et al. 2017). The combination of research, participation, and education is also central to the design-based implementation research described earlier (Penuel et al. 2011). Although community-based participatory research has its own challenges beyond the scope of this article, involving individuals most impacted by the research can improve the scientific process itself (Balazs and Morello-Frosch 2013), aid in decolonization of knowledge (Hall and Tandon 2017), and promote positive intervention outcomes (Minkler et al. 2017). Some researchers have published their methods of increasing campus–community engagement (e.g., Barnes et al. 2009), which may serve as a starting point for getting started with community-based participatory research in an academic setting.

## Conclusion

In this tutorial review, we have proposed a novel model designed to help use-inspired cognitive scientists identify opportunities for better translating cognitive science to classroom practice in higher education. With this model, we introduce cognitive scientists to implementation science—a field of research dedicated to systematically investigating the variability and unpredictability inherent in using cognitive-science-based interventions in real higher education contexts (Kelly 2012). To improve teaching and learning in higher education, researchers must provide practically useful information to instructors. Teaching is a complex endeavor affected by institutional, instructor, and student characteristics. Implementation science is the only formal research approach specifically designed to assess the way these characteristics impact use of educational interventions. As such, we have also made four concrete suggestions for how cognitive scientists could get their feet wet with implementation science:

1. conceptualize instructors as scientist–educators,
2. use pragmatic-controlled trials in research designs,
3. adopt and adapt planning and evaluation frameworks to this field, and
4. expand and improve transparency of reporting.

Under a research framework that includes implementation science, it is clear how and to what extent different research approaches contribute to the goal of scholarly teaching. By developing, supporting, and maintaining an implementation science in teaching and learning in higher education, scientists and educators may together move promising principles from cognitive science to use in real college classrooms with a pace and scope necessary to meet students’ needs.

## Data Availability

Not Applicable.
